# Natural Solutions to Antimicrobial Resistance: The Role of Essential Oils in Poultry Meat Preservation with Focus on Gram-Negative Bacteria

**DOI:** 10.3390/foods13233905

**Published:** 2024-12-03

**Authors:** Zorana Kovačević, Ivana Čabarkapa, Ljubiša Šarić, Marko Pajić, Dragana Tomanić, Bojana Kokić, Dragana D. Božić

**Affiliations:** 1 Department of Veterinary Medicine, Faculty of Agriculture, University of Novi Sad, 21000 Novi Sad, Serbia; zorana.kovacevic@polj.edu.rs; 2 Institute of Food Technology, University of Novi Sad, 21000 Novi Sad, Serbia; ljubisa.saric@fins.uns.ac.rs (L.Š.); dragana.tomanic@fins.uns.ac.rs (D.T.); bojana.kokic@fins.uns.ac.rs (B.K.); 3 Department for Epizootiology, Clinical Diagnostic, Pathology and DDD, Scientific Veterinary Institute Novi Sad, 21000 Novi Sad, Serbia; markopajic@niv.ns.ac.rs; 4 Department of Microbiology and Immunology, Faculty of Pharmacy, University of Belgrade, 11221 Belgrade, Serbia; dragana.bozic@pharmacy.bg.ac.rs

**Keywords:** antimicrobial resistance, multidrug resistant bacteria, poultry meat preservation, essential oils

## Abstract

The increase in antimicrobial resistance (AMR) is a major global health problem with implications on human and veterinary medicine, as well as food production. In the poultry industry, the overuse and misuse of antimicrobials has led to the development of resistant or multi-drug resistant (MDR) strains of bacteria such as *Salmonella* spp., *Escherichia coli* and *Campylobacter* spp., which pose a serious risk to meat safety and public health. The genetic transfer of resistance elements between poultry MDR bacteria and human pathogens further exacerbates the AMR crisis and highlights the urgent need for action. Traditional methods of preserving poultry meat, often based on synthetic chemicals, are increasingly being questioned due to their potential impact on human health and the environment. This situation has led to a shift towards natural, sustainable alternatives, such as plant-derived compounds, for meat preservation. Essential oils (EOs) have emerged as promising natural preservatives in the poultry meat industry offering a potential solution to the growing AMR problem by possessing inherent antimicrobial properties making them effective against a broad spectrum of pathogens. Their use in the preservation of poultry meat not only extends shelf life, but also reduces reliance on synthetic preservatives and antibiotics, which contribute significantly to AMR. The unique chemical composition of EOs, that contains a large number of different active compounds, minimizes the risk of bacteria developing resistance. Recent advances in nano-encapsulation technology have further improved the stability, bioavailability and efficacy of EOs, making them more suitable for commercial use. Hence, in this manuscript, the recent literature on the mechanisms of AMR in the most important Gram-negative poultry pathogens and antimicrobial properties of EOs on these meat isolates was reviewed. Additionally, chemical composition, extraction methods of EOs were discussed, as well as future directions of EOs as natural food preservatives. In conclusion, by integrating EOs into poultry meat preservation strategies, the industry can adopt more sustainable and health-conscious practices and ultimately contribute to global efforts to combat AMR.

## 1. Introduction

The increasing prevalence of antimicrobial resistance (AMR) has become a critical global health concern, not only in human medicine but also in the veterinary and food production sectors [1,2]. Each year, bacterial infections are responsible for an estimated 7.7 million deaths of people, with 4.95 million cases involving drug-resistant pathogens, and 1.27 million deaths directly caused by antibiotic-resistant bacteria [3]. Beside transmission of resistant genes among people, animals and environment, the widespread use of antibiotics in animal farming are main drivers on AMR spread and development [4,5,6,7,8,9,10]. Overuse and misuse of antimicrobials, including those critically important drugs for treating human disease has contributed to the emergence of resistant strains of bacteria, raising alarms about food safety and public health [11,12,13].

The food animal industry will encounter growing challenges in boosting productivity while minimizing AMR. This is particularly significant as the global demand for animal protein continues to rise, and food animals, since it has been estimated that by 2050, the global human populace is predicted to reach approximately 9.8 billion [14]. The poultry represents an important livestock sector worldwide [15], and play a crucial role in the global food supply [16]. Actually, poultry meat production worldwide has increased over the last 60 years and represents about one-third of the total global meat production [16]. Moreover, global poultry meat output is forecast to reach 146 million tons in 2024, up 0.8 percent year on year, principally driven by high international demand and lower feed costs, although highly pathogenic avian influenza outbreaks remain a challenge [17]. In the poultry industry, AMR poses significant risks, as the overuse and misuse of antimicrobial agents have led to the development of resistant strains of bacteria that can compromise meat safety and public health. There is evidence suggesting that the use of antimicrobials in animal food production is directly linked to the rise in AMR [18,19,20]; even alternatives gave promising results, in terms of clinical outcomes, as well as cost–benefit analyses [21,22]. Consequently, there has been a shift towards exploring natural, sustainable alternatives, such as plant-derived compounds, to enhance meat preservation and extend shelf life. In addition, essential oils (EOs) are generally recognized as safe (GRAS) by food safety authorities, making them suitable for direct application in food products [23]. Furthermore, traditional preservation methods, which often rely on synthetic chemicals, are increasingly scrutinized due to their potential impact on human health and the environment.

EOs have gained attention as natural antimicrobial agents, especially due to their potential effectiveness against gram-negative bacteria such as *Escherichia coli*, *Salmonella* spp., and *Campylobacter* spp. [24,25]. These pathogens are frequently implicated in foodborne illnesses, and their resistance to conventional antimicrobials is a growing concern in food safety and public health [26,27]. EOs offer a promising solution due to their complex chemical composition, which disrupts multiple bacterial mechanisms and potentially reduces the risk of resistance development compared to traditional antimicrobials [28,29]. Key components contributing to EOs’ antimicrobial activity are phenolic compounds (such as thymol and carvacrol), aldehydes (such as cinnamaldehyde), and terpenes (such as eugenol and limonene) [30]. These compounds act by disrupting bacterial cell membranes, denaturing proteins, and interfering with enzyme activity, which collectively results in bacterial cell death [31,32]. Research has shown that these mechanisms are particularly effective in preserving the microbiological quality of poultry meat by inhibiting the growth of spoilage organisms and foodborne pathogens [33]. While synthetic antimicrobials often involve energy-intensive production processes with byproducts that may contribute to environmental pollution, EOs are derived from renewable plant sources, offering a more sustainable foundation for antimicrobial solution [34].

Current solutions to AMR, including physical treatments like irradiation and chemical preservatives, are often limited by factors such as cost, consumer acceptance, and the potential for adverse effects on the food quality [35,36]. Synthetic additives may alter the sensory characteristics of food, which is undesirable from a market standpoint, and can be associated with adverse health implications [37]. Consequently, these conventional solutions have proven insufficient in fully addressing AMR concerns, necessitating the exploration of more sustainable and consumer-friendly alternatives.

This review aims to explore and give a comprehensive overview of the role of EOs in combating AMR and poultry meat preservation. It will cover the current state of research on the antimicrobial efficacy of EOs compounds, their mechanisms of action, and examine the practical challenges associated with their application. Furthermore, the review will address key factors influencing the effectiveness of EOs, including extraction methods, stability, and sensory impacts, and will explore future directions for research and development in this field. In addition, special focus will be on how these natural extracts can contribute to combating AMR while maintaining the quality and safety of poultry meat products.

## 2. Antimicrobial Resistance and Poultry Meat Preservation

As one of the most treating global health issues, AMR is affecting food production industries, including poultry. The poultry industry, as a crucial contributor and key player to the global food supply, is especially susceptible to the impacts of AMR. The widespread use of antimicrobials in poultry farming, both for therapeutic and prophylactic purposes, has led to the emergence and spread of drug-resistant bacterial strains [38,39,40,41,42,43]. The use of antibiotics in livestock and poultry has grown substantially, surpassing antibiotic consumption for medicinal purposes in humans. Indirect exposure to antibiotics through food products such as meat and milk has also risen, becoming more prevalent than direct consumption [13]. Furthermore, the misuse and overuse of antibiotics in poultry production, often involving subtherapeutic doses for growth promotion or disease prevention, have contributed to the selection of resistant bacteria, especially Gram-negative bacteria such as *Salmonella*, *Campylobacter* and *E. coli*. These resistant strains not only compromise the health of the poultry population but can also be transmitted to humans through the food chain, direct contact, or the environment [38,44,45,46]. This poses a significant challenge to meat safety, public health, and sustainability within the food supply chain. In addition, the genetic transfer of resistance elements between bacteria in poultry and human pathogens exacerbates the AMR crisis, highlighting the need for urgent action.

Poultry meat, being one of the most widely consumed animal proteins globally, is a significant vector for the transmission of AMR pathogens [47]. Hence, ensuring the microbiological safety of poultry meat has become a critical priority. Traditional methods for poultry meat preservation often rely on the use of synthetic chemicals and antimicrobials to control the growth of spoilage and pathogenic microorganisms and to inhibit the process of lipid oxidation extending the shelf-life, quality and safety of food products [48]. However, the overuse of these chemical preservatives has raised concerns about their long-term effects on human health, as well as their role in further promoting AMR. To address these concerns, there is growing interest in exploring natural alternatives for poultry meat preservation, with plant-derived compounds emerging as a promising solution. Hence, to reduce the use of traditional preservation and processing methods, bio-preservatives have been introduced. These are naturally occurring substances derived from plants, animals, and microorganisms that extend the shelf life of food products [49]. Furthermore, the antimicrobial properties of EOs derived from many plants have been recognized, leading to an interesting strategy for extending the shelf life of poultry meat and meat products by inhibiting the growth of spoilage and pathogenic microorganisms in meat while reducing the reliance on synthetic preservatives [50]. In addition, the use of plant-based preservatives aligns with the global movement towards sustainable and safer food production practices [51]. Moreover, the shift toward natural preservation methods is gaining momentum due to consumer demand for cleaner, more natural food products free from artificial additives [52,53]. In this context, EOs present a dual benefit; they not only help combat AMR but also meet the expectations of an increasingly health-conscious market. Finally, EOs emerge as a promising natural alternative, distinguished by their complex chemical compositions and broad-spectrum antimicrobial activity. In contrast to single-target synthetic antimicrobials, the multi-compound nature of EOs enables them to act through various mechanisms, such as disrupting bacterial cell membranes, interfering with enzyme activity, and inhibiting cellular processes in bacteria [54]. This multifaceted approach not only enhances their effectiveness but also reduces the likelihood of bacteria developing resistance. Furthermore, studies have shown that the presence of EOs can reduce the frequency of horizontal gene transfer, which is a major pathway through which antibiotic resistance genes spread among bacteria [55,56]. EOs disrupt bacterial communication systems, such as quorum sensing, which are crucial for biofilm formation and gene transfer [57]. By inhibiting quorum sensing, EOs may lower the rates at which resistance genes are exchanged, thereby reducing the spread of resistance within bacterial populations.

### 2.1. Mechanisms of Antimicrobial Resistance in the Most Important Gram-Negative Poultry Pathogens

#### 2.1.1. *Salmonella* spp.

The most important disease in humans caused by *Salmonella* spp. isolated from poultry is salmonellosis, which manifests through a range of symptoms including diarrhea, abdominal cramps, nausea, vomiting and fever. The disease is usually transmitted by the consumption of contaminated poultry or poultry products (e.g., raw/undercooked meat or eggs), by contact with contaminated surfaces or environments where poultry has been kept, or by handling infected poultry. The disease can take a severe course with a fatal outcome or a chronic course with asymptomatic transmission in immunocompromised patients, children and the elderly [58]. This disease is usually caused by non-typhoidal *Salmonella enterica* subspecies *enterica* serovars such as *Salmonella* Enteritidis and *Salmonella* Typhimurium [59], which are most prevalent isolates in raw chicken meat, although other serotypes that can also cause infections in humans, such as *Salmonella* Heidelberg, *Salmonella* Javiana, *Salmonella* Infantis, and *Salmonella* Thompson, are becoming more prevalent in the poultry industry [60]. Antibiotics used in poultry prophylaxis/metaphylaxis to control and prevent the spread of Gram-negative bacteria, including *Salmonella* spp. infections, within a flock may vary depending on regional regulations, the specific strain of *Salmonella* spp. and its resistance patterns. The most commonly used antibiotics are fluoroquinolones (enrofloxacin), macrolides (tyclosin), tetracyclines (oxitetracycline), sulfonamides and trimethoprim combinations (sulfamethoxazole-trimethoprim), aminogycosides (gentamicin and beta-lactams (amoxicillin) [61]. Although the use of antibiotics in poultry is subject to strict regulations, the use of antibiotics for growth promotion is banned in many regions and metaphylactic use is carefully controlled and monitored, this remains the main cause of the development of antimicrobial resistance. *Salmonella* spp. have developed several mechanisms of AMR, of which enzymatic inactivation, efflux pumps, modification of target molecules, and reduced permeability are the most important [38,60,62]. Beta-lactamase and aminoglycoside-modifying enzymes are the most frequently produced enzymes. The most important β-lactamases produced by *Salmonella* spp. include extended-spectrum β-lactamases (ESBLs), AmpC β-lactamases, and carbapenemases [63]. ESBLs hydrolyze penicillins, most cephalosporins (up to the third generation) and monobactams (aztreonam), and they are usually inhibited by β-lactamase inhibitors such as clavulanic acid [64]. One of the most common ESBLs found in *Salmonella* spp. is CTX-M, with several subtypes such as CTX-M-1, CTX-M-2, and CTX-M-91. Other ESBLs such as TEM and SHV are older ESBL types found in *Salmonella* spp., although they are less common compared to CTX-M1. AmpC β-lactamases hydrolyze a broader spectrum of cephalosporins (including cephamycins such as cefoxitin), penicillins, and monobactams, but are not inhibited by β-lactamase inhibitors such as clavulanic acid. AmpC genes can be found on the bacterial chromosome or on plasmids, which may facilitate horizontal gene transfer between bacteria. Although less common, some *Salmonella* spp. strains produce carbapenemases, such as KPC (*Klebsiella pneumoniae* carbapenemase) and NDM (New Delhi metallo-β-lactamase), which confer resistance to carbapenems [65].

The three main types of aminoglycoside-modifying enzymes (AMEs) found in *Salmonella* spp. are N-acetyltransferases (AACs), O-adenyltransferases (ANTs), and O-phosphotransferases (APHs). These enzymes chemically alter the structure of the antibiotic and reduce its ability to bind to bacterial ribosomes, thereby inhibiting protein synthesis [66]. AACs use acetyl-coenzyme A to acetylate the amino group at positions 3, 2′, and 6′ of the aminoglycoside molecule (enzymes AAC(3), AAC(2′), and AAC(6′)). The most commonly affected aminoglycosides are amikacin, gentamicin, and tobramycin. ANTs use ATP to transfer an adenyl group to the hydroxyl group of the aminoglycoside at positions 2′′, 3′′, and 4′ (enzymes ANT(2′′), ANT(3′′), and ANT(4′)). This modification leads to resistance to aminoglycosides such as streptomycin, kanamycin, and tobramycin. APHs use ATP to phosphorylate the hydroxyl group of the aminoglycoside at positions 3′, 2′′, and 6 (enzymes APH(3′), APH(2′′), and APH(6). Kanamycin, neomycin, and gentamicin are usually modified by these enzymes [67,68].

Efflux pumps play an important role in mediating multi-drug resistance (MDR) of *Salmonella* spp. as they actively efflux a broad spectrum of antibiotics and other toxic substances from the bacterial cell, thus reducing the intracellular concentration of the drugs. In *Salmonella* spp. there are several main groups of efflux pumps that belong to different families based on their structure and mechanism of action: the Resistance-Nodulation-Division (RND) family, Major Facilitator Superfamily (MFS), Small Multidrug Resistance (SMR) family, Multidrug and Toxic Compound Extrusion (MATE) family, and ATP-Binding Cassette (ABC) family [64]. Among them, the RND family plays an important role in MDR. The best characterized efflux pump in *Salmonella* spp., AcrA/B-TolC, which consists of a periplasmic adaptor protein (AcrA), an inner membrane transporter (AcrB) and an outer membrane channel (TolC), belongs to the RND family. It expels a wide range of antibiotics, including β-lactams, tetracyclines, chloramphenicoland fluoroquinolones [69]. MdsA/B-TolC is another RND-type efflux pump that is specific to *Salmonella* spp. and also contributes to antibiotic resistance [69,70]. Other important efflux pumps in *Salmonella* spp. are MdfA (MFS group), which effluxes several drugs, including aminoglycosides and macrolides; EmrE (SMR family), which is involved in the efflux of various toxic compounds and antibiotics; NorM (MATE family), which effluxes a range of drugs including fluoroquinolones and cationic dyes, and MacAB-TolC (ABC family), which utilizes ATP for the efflux of macrolides and other antibiotics [71]. Expression of efflux pump genes can be regulated by global transcriptional regulators such as MarA, SoxS and Rob, which respond to environmental stress and antibiotic exposure. Efflux pump genes can reside on mobile genetic elementssuch as plasmids, which facilitates the horizontal transfer of resistance between different bacterial species [72].

Resistance mediated by the modification of target molecules occurs against β-lactam antibiotics and fluoroquinolones. Alterations in penicillin-binding proteins (PBPs) are an important mechanism by which *Salmonella* spp. develop resistance to β-lactam antibiotics. PBPs are essential enzymes involved in the synthesis of bacterial cell walls, and β-lactam antibiotics target these proteins to inhibit cell wall synthesis. PBP-mediated resistance is the result of point mutations, overproduction of PBPs or acquisition of novel PBPs. Point mutations lead to reduced binding affinity or increased hydrolysis of β-lactams. Point mutations in PBPs can reduce the binding affinity of β-lactam antibiotics, making them less effective. Mutations in PBP3, PBP4 and PBP6 have been shown to lead to increased resistance to penicillin G and other β-lactams [73]. Some mutations can make the active site of PBPs more accessible to water, allowing hydrolysis of the antibiotic and reducing its efficacy. Overproduction of certain PBPs can compensate for the inhibition caused by β-lactam antibiotics, allowing the bacteria to continue synthesizing their cell walls. *Salmonella* spp. can acquire genes encoding novel PBPs with lower affinity for β-lactams through horizontal gene transfer. This can lead to the emergence of highly resistant strains [64]. Chromosomally mediated resistance to fuoroquinolones in *Salmonella* spp. is often accompanied by changes in the target molecules, in particular the enzymes DNA gyrase and topoisomerase IV. Quinolones penetrate the bacteria via porins and bind to the gyrase/topoisomerase IV-DNA complex, inhibiting replication and exerting a bacteriostatic effect. The resistance is caused by point mutations in the regions of the topoisomerase genes (*parC* and *parE*) and the DNA gyrase genes (*gyrA* and *gyrB*) that determine quinolone resistance. These mutations alter the protein structure of the binding sites, reduce their affinity for the drug, and block the lethal effect of fluoroquinolones and quinolones [74,75].

Another mechanism that contributes to AMR of *Salmonella* spp. is the reduced permeability of the outer membrane due to the presence of outer membrane proteins (OMPs), such as porins, and modifications of the outer membrane, such as changes in lipid composition. These mechanisms increase the barrier function of the membrane and reduce the uptake of antibiotics and their intracellular concentration [76]. Porins are channels that allow the passive diffusion of small hydrophilic molecules, including nutrients and antibiotics, into the bacterial cell and are essential for bacterial survival and pathogenicity. Like other Gram-negative bacteria, *Salmonella* spp. express two major porins, OmpC and OmpF. Altered expression of the porins or a change in their structure can reduce the uptake of antibiotics and thus lead to AMR. Reduced expression of the porins OmpC and OmpF is associated with increased resistance to β-lactams and fluoroquinolones [77]. The expression of OMPs is regulated by two-component systems such as EnvZ/OmpR, which respond to environmental signals. The EnvZ/OmpR regulatory systems can alter the expression of OMPs in response to antibiotic exposure and increase resistance to β-lactams by reducing outer membrane permeability [78]. *Salmonella* spp. also express the porins OmpD and OmpW, the altered expression of which can also contribute to antibiotic resistance [64]. A reduction in the membrane permeability of *Salmonella* spp. occurs when genetic changes alter membrane proteins that affect the pores of the transport system and block the entry of antibiotics. In polymyxin resistance, alterations in the lipid A part of the lipopolysaccharide involving phosphoethanolamine and 4-amino-4-deoxy-L-arabinose reduce the negative charge of the membrane and its affinity for polymyxin [60].

#### 2.1.2. *Escherichia coli*

Avian pathogenic *E. coli* (APEC), which causes respiratory and systemic infections such as colibacillosis in poultry, can also cause disease in humans. These infections are usually caused by extraintestinal pathogenic *E. coli* (ExPEC) strains, which include APEC. In humans, ExPEC can cause urinary tract infections (UTI), neonatal meningitis and septicemia. Although direct transmission from poultry to humans is rare, it is possible, especially in individuals with compromised immune systems or in close contact with infected birds [79].

The main AMR mechanisms of *E. coli* are similar to those of other enterobacteria, such as *Salmonella* spp. Antimicrobials are often inactivated by ESBLsand AmpC-β-lactamases, AMEs AAC, ANT, or APH and chloramphenicol acetyltransferase [80]. Carbapenemase-producing *E. coli* (VIM-1, NDM-1 and OXA-48 producers) have also been isolated from livestock (poultry, cattle and pigs) and their environment [81]. *E. coli* isolated from poultry usually has multiple efflux pumps that contribute to antimicrobial resistance. The primary efflux pump in *E. coli*, AcrAB-TolC, is responsible for the excretion of a broad spectrum of antibiotics, including tetracyclines, fluoroquinolones and β-lactams. Other efflux pumps include MdfA, which is responsible for resistance to several antibiotics, including chloramphenicol and fluoroquinolones, the EmrAB-TolC pump, which is involved in resistance to macrolides and other antibiotics, and the MacAB-TolC pump, which specifically excretes macrolides and contributes to resistance to these antibiotics [82,83]. Mutations in porin proteins such as OmpF and OmpC are often associated with antibiotic resistance in *E. coli*. Mutations in the OmpF porin can reduce the permeability of the outer membrane and thus limit the penetration of antibiotics such as β-lactams. In addition, overexpression of OmpX can lead to reduced expression of OmpC and OmpF, which in turn contributes to reduced susceptibility to antibiotics [84,85]. Several mutations of target proteins contribute to antimicrobial resistance in *E. coli*, with mutations of PBP3, PBP4 and PBP6 and mutations in the *gyrA* and *parC* genes being the most common. Resistance can also be mediated by mutations in ribosomal proteins or rRNA which confer resistance to antibiotics such as aminoglycosides and macrolides, as well as mutations in the dihydrofolate reductase gene *folA* which lead to resistance to trimethoprim by reducing the binding affinity of the drug [80,86].

#### 2.1.3. *Campylobacter* spp.

*Campylobacter jejuni* and *Campylobacter coli* isolated from poultry primarily cause campylobacteriosis in humans, a gastrointestinal disease characterized by diarrhea, abdominal cramps, fever, nausea and vomiting. In severe cases, it can lead to autoimmune complications such as Guillain-Barré syndrome, or fatal sepsis. Most infections are caused by the consumption of undercooked or contaminated poultry, but can also be caused by contact with infected animals or ingestion of contaminated water [87,88]. For many years, fluoroquinolones have been the preferred antibiotics for the treatment of campylobacteriosis. However, their extensive use both in the clinic and in animal husbandry has led to the development of fluoroquinolone-resistant strains of *Campylobacter* spp. Currently, macrolides are the antibiotics of first choice for the treatment of campylobacteriosis, although tylosin and erithromycin are still used as growth promoters in some poultry farms [89].

Similarly to the previously discussed Gram-negative bacteria, the main mechanisms of antimicrobial resistance in *Campylobacter* spp. are modification of target molecules, active efflux and enzyme inactivation [90,91,92,93,94]. The modification of target molecules leads to resistance to fluoroquinolones, macrolides and tetracyclines. A single-point mutation in the DNA gyrase gene (*gyrA*) leads to high-level resistance to quinolones and fluoroquinolones [95]. Similarly, modifications in the 23S rRNA gene and ribosomal proteins (L4 and L22) confer resistance to macrolides [91,96]. Mutations in the *tet(O)* gene, located on the conjugative plasmid, lead to binding of the TetO protein to its target, the ribosome A site, and its modification leads to ribosome protection [96]. The primary efflux pump in *Campylobacter* spp. is the CmeABC system, which consists of three components: CmeA, a periplasmic fusion protein, CmeB, which acts as inner membrane transporter, and CmeC, which is an outer membrane channel protein. Antibiotics affected with efflux are β-lactams, fluoroquinolones, macrolides and tetracyclines. In addition to CmeABC, *Campylobacter* spp. expresses at least nine other efflux pumps that contribute to antimicrobial resistance to various antimicrobial agents [61,95]. Resistance mediated by enzyme inactivation of antimicrobial agents is an important mechanism in the resistance of *Campylobacter* spp. to β-lactams and aminoglycosides. The *bla_OXA-__61_* gene plays a crucial role in the resistance of *Campylobacter* spp. to beta-lactam antibiotics. The β-lactamase enzyme OXA-6, which is produced by *bla_OXA-__61_*, can hydrolyze a number of beta-lactam antibiotics, including amoxicillin, ampicillin and ticarcillin. Mutations in the promoter region of *bla_OXA-__61_* can further enhance its expression, leading to higher levels of resistance [97]. Several genes encoding the AMEs AAC, APH and ANT are involved in resistance to aminoglycosides such as gentamicin. These genes are located on mobile genetic elements such as multidrug-resistant plasmids, integrons and transposons and are easily transferable between different strains of *C. jejuni*, *C. coli* and other bacteria [90,98].

**Figure 1:**
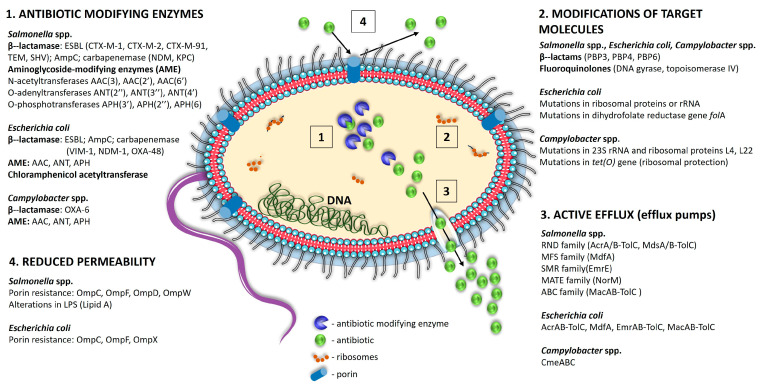
Major mechanisms of antimicrobial resistance in Gram-negative pathogens isolated from poultry meat.

The major mechanisms of antimicrobial resistance in *Salmonella* spp., *E. coli* and *Campylobacter* spp. are presented in Figure 1.

## 3. Antimicrobial Properties of Essential Oils Against the Most Important Gram-Negative Poultry Meat Isolates

The antimicrobial properties of EOs derived from many plants have been recognized, what enables them to become an interesting strategy to extend the shelf life of meat and meat products [99]. Certain EOs and their volatiles may work as natural food preservatives compared to synthetic and chemical preservatives with less toxicity [100]. Beside low toxicity, EOs are becoming potential antibiotic alternatives due to their natural origin and lack of residues [101]. Moreover, extraction technique plays an important role, which has led to improve conventional techniques in favor of green emerging technologies that allow to preserve better target bioactive components, operating at lower temperatures and avoiding as much as possible the use of solvents, with more sustainable processing and reduced energy use and environmental pollution [102]. Studies suggest that EOs have a lower environmental persistence, making them less likely to contribute to AMR through environmental contamination [103]. This characteristic is especially valuable in the context of meat preservation, where residual antimicrobials can enter wastewater and potentially impact microbial populations in natural ecosystem [10].

EOs have been identified as beneficial alternatives to synthetic antioxidants in meat and meat products, so they can be used alone or be combined with other EOs, food additives or preservation techniques, to improve the shelf life of meat and meat products [104,105,106]. Actually, EOs were considered a good alternative and safe for consumer use because the majority of EOs are generally recognized as safe (GRAS) status by the Food and Drug Administration (FDA) [23].

As mentioned above, poultry meat is highly susceptible to microbial contamination by Gram negative pathogens such as *Escherichia coli*, *Salmonella* spp., and *Campylobacter* spp., which are commonly associated with foodborne illnesses. On the other hand, studies have shown that EOs derived from plants like thyme (*Thymus vulgaris*), oregano (*Origanum vulgare*), clove (*Syzygium aromaticum*), and rosemary (*Rosmarinus officinalis*) demonstrate strong antimicrobial activity against these pathogens by targeting microbial cell walls, disrupting membrane integrity, and interfering with cellular metabolism [102,107]. Beside composition, factors determining the activity of EOs are functional groups present in active components, and their synergistic interactions [108].

EOs exhibit several well-documented antibacterial mechanisms of action that lead to the inhibition of bacterial growth [33,109,110]. The antimicrobial efficacy of EOs largely depends on their concentration and the presence of key bioactive compounds, such as carvacrol, thymol, eugenol, and linalool, which exhibit synergistic effects when combined [111,112]. Once extracted, these compounds display greater inhibition of Gram-positive than Gram-negative bacteria due to membrane disruption as the main mechanism of action involved [102].

Actually, the primary actions involve disrupting microbial cell membranes due to the lipophilic nature of EOs, which allows them to integrate into and destabilize lipid bilayers, leading to increased cell permeability and leakage of essential intracellular components [113,114,115]. For instance, carvacrol and thymol, prominent components in thyme and oregano EOs, are known to destabilize bacterial cell membranes, leading to leakage of cellular contents and eventual cell death [116,117,118].

Additionally, the antibacterial activity of cinnamon and oregano EOs is mainly attributed to its high carvacrol and thymol content that can disintegrate Gram-negative bacteria’s outer membrane, releasing lipopolysaccharides increasing the permeability of the cytoplasmic membrane to ATP [119]. Probably, phenolic compounds are the mainly responsible for their microbial activity linked to the position of their hydroxyl group [120,121]. Therefore, in general, the more phenolic compounds the more antibacterial properties against foodborne pathogens [122].

Furthermore, bioactive compounds can penetrate biofilms, a major defense mechanism of bacteria, thereby enhancing their effectiveness against biofilm-forming pathogens often found on poultry surfaces [123,124].

On the other hand, sometimes it is necessary to use EOs in higher concentrations to achieve the same effect as commercial additives, which could cause undesirable effects on the product or even toxicity problems [125]. This makes necessary to consider their use combined with other preservatives, looking for a synergistic effect that allows to achieve a better activity at a lower concentration but sufficient to avoid the appearance of undesirable effects [104]. For example, rosemary EO in concentrations higher than 0.2% were not acceptable because of the organoleptic properties they imparted to the poultry meat [126]. Hence, it is very challenging to find a compromise between effective doses of flavoring agents like EOs and sensory acceptability of the flavored food, physicochemical properties of meat products present the factors that influence the consumers’ overall acceptability and provide the base for the final judgment of some treatment’s applicability [127].

Addition of EOs and their volatile components can be used against pathogenic bacteria of *Salmonella* species which will serve as green preservatives to food industry [100]. Antimicrobial effect of thyme, oregano and lemon EOs on multidrug-resistant *Salmonella* Typhimurium in chicken filets was assed where a significant reduction of *S*. Typhimurium counts with highest inhibition obtained using 1% lemon EO [128]. The sensory properties of treated chicken filets were improved by all used EOs, compared to the control samples after 6th and 8th day of the storage period [128].

The study conducted on antibiotic-resistant *Salmonella* spp. tested the antimicrobial activity of 13 lactic acid bacteria strains and two EOs (*Thymus vulgaris* and *Origanum vulgare*), and their compositions for the purpose of inhibiting [129]. The most effective composition for the control of *Salmonella* tested in this study consists of thyme EO (1.0%) with the following LAB strains: LUHS122, LUHS242, LUHS210, LUHS244, LUHS135, LUHS71, and LUHS245 [129]. Another study tested the in vitro antimicrobial activity of EOs against *Salmonella enterica* serotypes Enteritidis and *S.* Typhimurium strains isolated from poultry [130]. Among tested EOs (*Aloysia triphylla*, *Cinnamomum zeylanicum*, *Cymbopogon citratus*, *Litsea cubeba*, *Mentha piperita*, *Syzygium aromaticum*) the highest antibacterial activity was observed for *C. zeylanicum* (minimum inhibitory concentrations (MICs) ranging from 1.26 mg/mL to 0.63 mg/mL), *S. aromaticum* (MICs from 2.637 mg/mL to 0.164 mg/mL) and the mixture (MICs from 1.289 mg/mL to 0.322 mg/mL) [130].

Furthermore, study focused to the efficiency of various plant metabolites against *Salmonella* species in food models, were found for carvacrol (EO component) in gaseous phase, can reduce or eliminate *S.* Enteritidis in artificially inoculated raw chicken meat [131]. Moreover, the results showed that adding 0.2% rosemary (*Rosmarinus officinalis* L.) EO to poultry filets did not reduce the size of the population of *S.* Typhimurium and *L. monocytogenes*, while it significantly decreased the lightness value and increased the redness value of stored filets. Moreover, combination with modified-atmosphere packaging reduced the level of lipid oxidation indicating that in a suitable combination, rosemary EO can be applied to improve the quality of meat [126].

EOs of *Melaleuca alternifolia* (tea tree) and *Thymus vulgaris* (thyme), of the family *Myrtaceae* and *Lamiaceae* respectively, were assessed for antibacterial activity against different avian pathogens isolated from infected chickens which involved *E. coli*, and *Salmonella Gallinarum* [132]. Thyme EO exhibited higher activity than tea tree EO against both pathogens. Mean inhibition zones, MIC and MBC values of bacterial strains varied from 19 and 34 mm, 0.03–0.15% to 0.07–0.3%, respectively. GC-MS analysis of thyme EOs showed the presence of 13 components where the major components were carvacrol, thymol, terpinene-4-ol, α-Terpinene, carvacrol methyl ether. Moreover, tea tree EOs showed the presence of 16 components upon GC-MS analysis where the major compounds were Limonene, γ -Terpinene, αTerpinene, Cineol and α-Terpinolene [132].

The results of study where the antibacterial and anti-biofilm activity of summer savory (*S. hortensis*) EO against *E. coli* and *Salmonella* isolated from poultry were tested, this EO showed the growth inhibition and bactericidal activity against these pathogens. Moreover, this study demonstrated anti-biofilm activity of *S. hortensis* EO against both tested bacteria [133].

In addition, the in vitro antimicrobial activity of EOs and spice powders of cumin, black seeds, cloves, cinnamon, and marjoram were tested against *E. coli* O157 isolates from raw and processed poultry meat samples showing a significant decrease in microbial count in treated samples either with the spices powder EOs of the tested medicinal plants compared to control samples during storage time period [134]. In study where in vitro antimicrobial efficacy was tested on sixteen EOs against *E. coli* isolated from poultry MIC determination found good anti-*E. coli* activity with *C. zeylanicum* (2.52 mg/mL), *C. citratus* (1.118 mg/mL), *L. cubeba* (1.106 mg/mL), *M. piperita* (1.14 mg/mL) and *S. aromaticum* (1.318 mg/mL) EOs [135]. Additionally, one hundred and ninety-one compounds were identified in the tested EOs and mixtures [135].

Also, in case where thyme, rosemary or clove EOs at 1.5% were added to mechanically deboned chicken meat protein films, antioxidant and antimicrobial properties were significantly improved with EOs, whereas the films containing clove EO showed higher antimicrobial activity against *E. coli*, while the films containing thyme EO showed higher activity against *L. monocytogenes* [136].

Moreover, rosemary EO, mint EO, nisin and lactic acid were incorporated at 0.5% to develop a novel functional packaging film including chitosan pectin and starch polymers (0.75:1.5:0.75 *w*/*w*) where the incorporation of rosemary EO and nisin exhibited the highest inhibitory activity against all tested pathogenic strains (*Bacillus subtilis*, *E. coli*, and *Listeria monocytogenes*) [137]. This study validated that incorporation of natural additives in active biocomposite films offers promising functional ingredients for packaging materials for various food applications.

Along with *E. coli* O157:H7 and *Salmonella* spp., *Campylobacter* spp. pathogenic strains could put food safety at risk since they are not eliminated with heat treatments to which the products are sometimes subjected [102]. Since *Campylobacter jejuni* mainly contaminates the surface of meats and meat products, there is an imperative need to employ antimicrobial packaging to suppress the reproduction of *C. jejuni* on meat surface [138]. When it comes to EOs application, multiple mechanisms are responsible for the effect of EOs against pathogens including *Campylobacter* [139]. As a consequence, the impact of EO blends on *Campylobacter* populations may vary considerably in pre- and post-harvest applications [140].

Both EOs from *Origanum syriacum* (98%) and *O. ehrenbergii* (97.3%) showed promising potential in inhibiting the growth of the tested *E. coli* strains isolated from raw chicken meat [141]. EO from *O. syriacum* exhibited superior efficacy against 200 *E. coli* strains, inhibiting 46.1% at 200 mg/L and all at 400 mg/L, while *O. ehrenbergii* EO showed slightly lower inhibition, affecting 41.6% at 200 mg/L and all at 400 mg/L [141].

Laurel (*Laurus nobilis*) EO, native of the Mediterranean area highly cultivated in Europe is characterized by its contents of 1,8-cineole, α-terpinenyl acetate, linalool, methyleugenol, sabinene and carvacrol, which could be responsible for their antimicrobial activity against foodborne spoilage and pathogenic bacteria [50]. Application of laurel EO in chicken meats packaged in microaerobic atmosphere has promising results against *C. jejuni* since as natural preservative, this EO allowed to protect meat products and extend their shelf life due to the reduction of *C. jejuni* counts, which displayed significant reductions of 6.14 log CFU/g at the end of storage compared to untreated samples (8.14 log CFU/g) [142]. Furthermore, a higher sensory quality was observed in treated samples, since its use gave rise to fresh meat odor for longer time.

Thyme EOs have proved to be more efficient than the usage of Coriander in the suppression of *C. jejuni* growth in minced chicken meat [143]. Actually, thyme EO (1 and 2%) decreased count of *C. jejuni* (CFU/g) from 3.8 × 10^7^ (initial load) to 7.3 × 10^5^, and 1.2 × 10^3^ with reduction percentages 97.27% and 99.99% on 6th day of storage, respectively. Coriander EO (1 and 2%) decreased count of *C. jejuni* (CFU/g) to 3.8 × 10^6^ and 9.5 × 10^5^ with reduction percentages 85.00% and 96.27% on 6th day of storage, respectively [143].

The development of thyme EO nanoencapsulated in chitosan-gelatin nanofibers could be used as an alternative to nitrites in meat products [102]. In order to control the propagation of *C. jejuni*, the gelatin nanofibers containing thyme EOs with β-cyclodextrin ε-polylysine nanoparticles had a promising prospect in chicken meat preservation due to reduction in the counts without impact on sensory evaluation [102]. Moreover, the potent anti-*Campylobacter* activity was proven in the formulations containing cinnamon, lemongrass, clove, geranium and oregano EOs (>50 mm) in nanostructured lipid carriers that inhibited the growth of *Campylobacter* spp. isolated from chicken carcasses at low concentrations (0.2 mg/mL) [144]. These results are summarized in the Supplementary Material (Table S1).

## 4. Main Chemical Compounds of Essential Oils with Antimicrobial Activity Against Gram-Negative Bacteria

**Figure 2:**
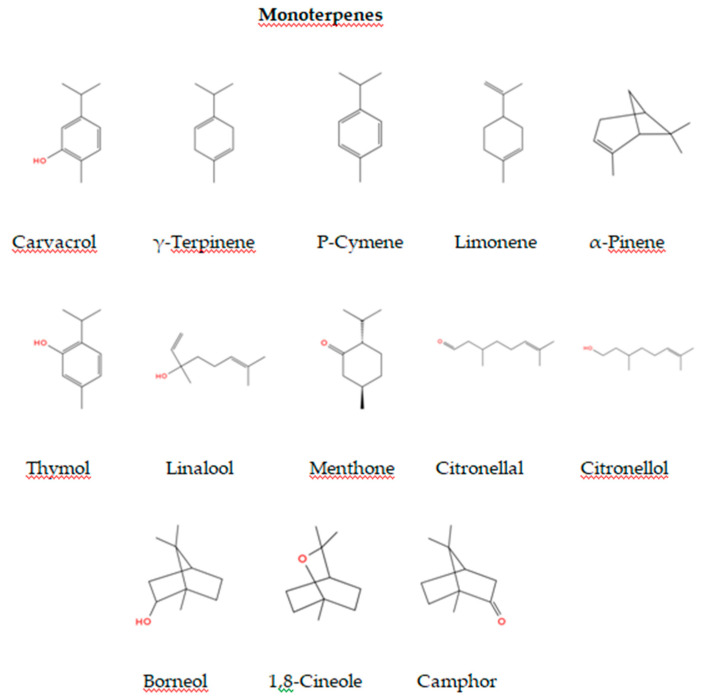
Chemical Structures of Key Monoterpene Components in EOs.

**Figure 3:**
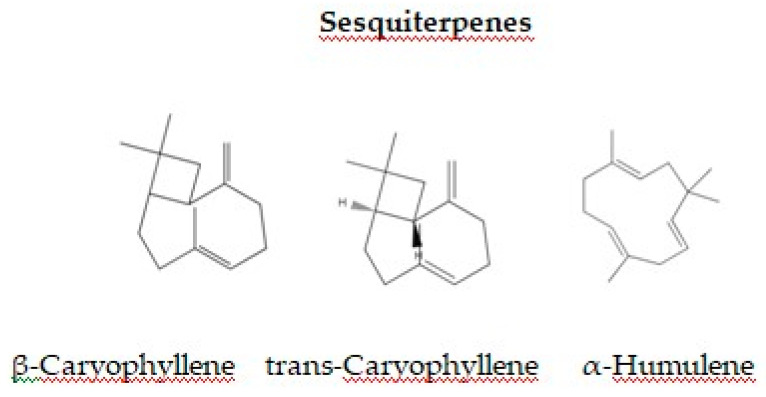
Chemical Structures of Key Sesquiterpene Components in EOs.

EOs contain a complex mixture of bioactive compounds, primarily terpenes and terpenoids, which exhibit strong antimicrobial properties. Among these, monoterpenes (Figure 2) and sesquiterpenes (Figure 3) are especially effective against Gram-negative bacteria including *E. coli*, *Salmonella* spp., and *Campylobacter* spp. [145].

It is mentioned that the chemical composition of the EOs determines their characteristics and therefore their mode of action. However, due to a great variety of compounds, their antimicrobial activity cannot be only attributed to a single mechanism of action [146]. Usually, EOs have higher antimicrobial activity than their major components, which suggests possible interactions between all of its constituents [147]. Although EOs have been empirically used as antimicrobial agents, their spectra of activity and mechanisms of action remain unknown for most of them [148].

Monoterpenes, including thymol, carvacrol, and linalool, are abundant in EOs from thyme, oregano, and rosemary [149]. These compounds are known for their broad-spectrum antimicrobial effects. Antimicrobial mechanism of carvacrol and thymol, which are the two major constituents of the EOs used in the poultry meat preservation, is based on their ability to disintegrate the outer membrane of Gram-negative bacteria, releasing lipopolysaccharides and increasing the permeability of the cytoplasmic membrane to ATP which are critical for bacterial metabolism and survival [150]. Furthermore, monoterpenes may induce oxidative stress within bacterial cells, causing irreversible damage to proteins and nucleic acids [114]. Additionally, linalool, as monoterpene alcohol poses antibacterial and antifungal properties [151], as well as antioxidant [152]. Its action primarily involves disrupting the integrity of bacterial cell membranes, increasing membrane permeability, which leads to leakage of essential intracellular components such as ions, nucleotides, and ATP. This disruption impairs cellular homeostasis and metabolic processes, ultimately leading to cell death [153]. Linalool can also interact with membrane proteins, affecting enzyme functions and interfering with the energy production pathways within microbial cells [154]. Moreover, linalool has shown the ability to induce oxidative stress by promoting the production of reactive oxygen species (ROS), further damaging bacterial cell components, including DNA, proteins, and lipids [155]. These combined effects contribute to linalool’s broad-spectrum antimicrobial efficacy against various pathogens, including Gram-negative bacteria.

The damage caused by certain monoterpenes, such as carvacrol and its precursor p-cymene, was evaluated on the biomembranes of *E. coli* indicating that the antimicrobial effects of p-cymene and carvacrol are likely due to disruptions in the lipid components of the bacterial membrane, leading to increased membrane permeability and loss of cellular integrity [156]. Additionally, the ways in which carvacrol and p-cymene impact protein synthesis and cell motility in *E. coli* O157:H7 strain were also recently investigated [157], further highlighting their potential to inhibit essential cellular processes in Gram-Negative bacteria. Antimicrobial activity against Gram-negative bacteria was proven in the study highlighting the bactericidal potential of limonene, a major constituent of EOs, against *E. coli*, with enhanced effectiveness at acidic pH (pH 4.0) compared to neutral pH (pH 7.0). The findings indicate that limonene targets β-sheet proteins and outer membrane structures in *E. coli*, suggesting its promising application as a natural preservative in food products, particularly when combined with mild heat treatments to improve microbial inactivation [158]. Furthermore, scanning electron microscopy results revealed that individual components of EOs such as citronellol altered the cellular morphology and destroyed the cell membrane confirming antibacterial activity [159].

Sesquiterpene component in EOs, such as *trans*-Caryophyllene possibly act as inhibitors of the NorA, Tet(K), MsrA, and MepA efflux pumps in resistant *Staphylococcus aureus* strains [160]. Despite this, further studies are required to describe the molecular targets involved in the action of these organic compounds. Other researchers have tested the antimicrobial potential of the sesquiterpene, β-caryophyllene, against various microorganisms, but they were unable to explain for the antibacterial mechanism with their study [161,162].

## 5. Studies Supporting the Antimicrobial Role of Essential Oils in Poultry Meat Preservation

To validate the theoretical discussions, this section highlights recent case studies and primary research that examine the application and efficacy of EOs as natural preservatives in poultry meat.

In a study by Porter et al. [163] white mustard EO and carvacrol were applied to poultry meat to assess their effectiveness in reducing microbial load and preserving meat quality over time. Results indicated that these EOs significantly inhibited the growth of *Salmonella* spp. in ground chicken stored at 4 and 10 °C, with minimal impact on the sensory qualities of the meat. This case illustrates the practical potential of these monoterpenes to act as natural preservatives in poultry meat. Furthermore, in the study where the effects of thyme and balm EOs where evaluated on the 3-wk storage of fresh chicken breast meat at 4 °C, Balm EO significantly limited the growth of *Salmonella* spp., whereas thyme EO effectively inhibited the growth of *E. coli* indicating that these 2 EOs effectively reduced deteriorative processes in chicken meat and extended the shelf life of this fresh product [164].

Sharma et al. [165] conducted research on the antimicrobial effectiveness of EOs under refrigeration, simulating commercial storage conditions for poultry. This study demonstrated that essential oils such clove oil (0.25%), holy basil oil (0.125%), cassia oil (0.25%) and thyme oil (0.125%) effectively extended the shelf life of chicken meat by inhibiting spoilage bacteria, including *Salmonella* spp., under cold storage. These findings provide experimental support for the use of EOs in commercial poultry preservation systems. Moreover, all vacuum packaged frozen fresh chicken sausages were found quite acceptable even at the end of storage period of 45 days after EOs (clove oil (0.25%), holy basil oil (0.125%), cassia oil (0.25%) and thyme oil (0.125%)) were incorporated in them [165]. One more study demonstrated that an edible film with oregano and thyme EOs effectively extends the shelf life and preserves the quality of chicken meat patties during refrigerated storage. The EO-treated patties showed lower microbial counts, reduced lipid oxidation, and stable pH levels compared to controls, maintaining acceptable sensory qualities up to 30 days [166]. This approach offers a natural, scalable alternative to synthetic preservatives in meat products.

Interestingly, the results suggest that the EO extracted from *C. cyminum* L. could be applied as an alternative natural preservative to control food-borne disease and have the potential for further development of new antibacterial agents since it has shown antibacterial activities against *Bacillus cereus*, *S. aureus*, *E. coli*, and *S.* Typhi [167].

## 6. Methods of Extraction and Application

The effectiveness of plant-derived bioactive compounds in preserving poultry meat largely depends on the extraction methods used. Several techniques are commonly employed to obtain these bioactive components, such as solvent extraction, steam distillation, supercritical fluid extraction and hydrodistillation using the Clevenger apparatus [168,169]. The hydrodistillation method is valued for its ability to produce high-purity oils without the need for chemical solvents, which aligns well with the demand for natural and eco-friendly extraction processes [170]. Additionally, it is suitable for both research and small-scale production, though it may require optimization when scaling up for industrial applications. Moreover, hydrodistillation with the Clevenger apparatus remains a benchmark in EOs extraction due to its simplicity and effectiveness, despite the development of newer techniques like supercritical fluid extraction [171].

Each with them have own advantages and limitations. Furthermore, replacing synthetic additives in meat products is a challenge, given their importance for sensory characteristics and food safety [172]. Actually, one of the primary limitations of using EOs as antimicrobial agents in the poultry meat preservation is their strong flavor and aroma, which can affect the sensory qualities of the product when applied in higher concentrations [102]. Addressing this challenge is critical for consumer acceptance and has prompted research into advanced techniques and formulations that enhance EO efficacy at lower, more palatable levels [34].

One of the most widely used methods is solvent extraction [173]. This method of extraction involves using organic solvents like ethanol, methanol, or acetone to isolate bioactive compounds from plant materials and is preferred due to its simplicity and ability to extract a wide range of compounds, including phenolics, flavonoids, and EOs [174]. However, the choice of solvent is critical as it can influence the yield and purity of the extracted compounds [175]. Another often used method for EOs is steam distillation that involves passing steam through plant material to vaporize volatile compounds, which are then condensed and collected. This method is particularly effective for extracting heat-stable, volatile bioactive compounds such as those found in herbs like thyme and oregano [176,177,178]. Besides its advantages, its main limitation is the potential degradation of some heat-sensitive components [179]. Furthermore, supercritical fluid extraction (SFE) uses supercritical carbon dioxide as a solvent to extract bioactive compounds from plant materials [180]. This technique is highly efficient and environmentally friendly, producing high-purity extracts without the use of toxic solvents [181]. SFE is particularly useful for extracting lipophilic compounds, such as EOs and carotenoids, but requires expensive equipment and specialized conditions [182,183].

Each extraction method affects the composition, concentration, and stability of the bioactive compounds, influencing their antimicrobial and antioxidant effectiveness when applied to poultry meat. Moreover, once extracted, plant bioactive compounds can be incorporated into poultry meat through various application methods, such as direct addition, edible coatings and films, encapsulation, as well as spraying or dipping. During the direct addition, plant extracts are directly mixed into poultry meat products during processing [184]. The extracts can be added in their pure form or incorporated into marinades, brines, or coatings to ensure uniform distribution throughout the product [185,186,187]. On the other hand, direct addition is a straightforward method, but achieving an even dispersion of the bioactive compounds may be challenging in larger meat cuts. Plant extracts can also be incorporated into edible coatings and films applied to the surface of poultry meat using edible coatings and films [188]. These coatings act as a barrier, reducing moisture loss and preventing microbial growth [189]. Edible films, made from natural materials like proteins, lipids, or polysaccharides, can be infused with antimicrobial and antioxidant plant extracts, offering a sustainable alternative to synthetic packaging materials [190,191]. Furthermore, to protect sensitive bioactive compounds from degradation and improve their controlled release, encapsulation techniques are often used. Encapsulation involves enclosing plant extracts within carriers such as liposomes, nanoparticles, or emulsions, which are then applied to poultry meat [192,193]. This method enhances the stability and bioavailability of plant-derived compounds, allowing for more effective preservation [194]. Moreover, encapsulation techniques, by including microencapsulation and nanoencapsulation allow EOs to be embedded within a carrier material, while the controlled-release approach stabilizes the EO compounds, preserving their antimicrobial properties over extended periods while reducing their direct contact with the meat surface, thereby minimizing flavor transfer [195]. Studies show that encapsulated EOs can maintain strong antimicrobial activity against pathogens such as *E. coli* and *Salmonella* spp. at lower concentrations, effectively reducing undesirable sensory impact [196]. Furthermore, advanced application methods, such as encapsulation and nanoemulsions, enhance the stability and efficacy of EOs, allowing them to be used in smaller quantities [197]. This not only improves cost efficiency but also reduces the overall environmental footprint by limiting the amount of EO required for effective antimicrobial action. Controlled-release systems, such as biodegradable films infused with EOs, have shown potential to gradually release antimicrobial compounds onto the meat surface, thereby prolonging their effectiveness and minimizing environmental waste [198].

Additionally, poultry meat can also be treated with plant extracts by spraying or dipping the meat into solutions containing the bioactive compounds [199,200]. This method ensures surface contact and is particularly effective for treating poultry skin, where microbial contamination is most likely to occur. Spraying or dipping is a cost-effective and simple method, but may offer limited protection to the interior of the meat. The choice of application method depends on factors such as the type of poultry product, the desired shelf-life extension, and the specific characteristics of the plant extract used. Proper application of these extracts can significantly enhance the preservation of poultry meat while minimizing the use of synthetic preservatives. In addition, availability and consistency in EO quality are critical for their adoption in large-scale poultry production [201]. This challenge could be mitigated by developing standardized production protocols and quality assurance processes to ensure that EOs meet consistent potency requirements for antimicrobial activity [202].

Furthermore, by collaborating with farmers and producers of EO raw materials, poultry producers can help stabilize EO availability and maintain a steady supply chain. By implementing these strategies, the use of EOs in commercial poultry production could become economically feasible and scalable, supporting a shift toward natural preservation methods aligned with sustainability and consumer preference trends. Additionally, widespread adoption of EOs in the poultry industry will also depend on regulatory frameworks that recognize and support their use as food preservatives. Clear guidelines on dosage, safety, and labeling will be vital to ensure consumer trust and compliance.

## 7. Conclusions

This manuscript highlighted that applying EOs to poultry meat can significantly reduce microbial loads of Gram negative foodborne bacteria, extend shelf life, and improve safety without compromising the sensory qualities of the meat when used at appropriate concentrations. While EOs are promising, challenges remain in optimizing their application, as high concentrations may affect meat flavor and aroma. Therefore, further studies are warranted to develop effective EO based formulations that ensure microbial safety, meet consumer acceptability, and maintain product quality in poultry meat products. Through innovative approaches such as encapsulation and edible coatings, EOs hold significant potential in advancing natural antimicrobial strategies within the poultry industry.

## Supplementary Materials

The following supporting information can be downloaded at: https://www.mdpi.com/article/10.3390/foods13233905/s1, Table S1: Antimicrobial Properties of EOs derived from aromatic plants against the most important Gram-negative poultry meat isolates.
