# Supplementary material for: Natural Solutions to Antimicrobial Resistance: The Role of Essential Oils in Poultry Meat Preservation with Focus on Gram-Negative Bacteria
Source: Foods. 2024 Dec 3;13(23):3905. doi: 10.3390/foods13233905 (PMC11640364; doi:10.3390/foods13233905)
Supplement: Supplementary file 1 [file foods-13-03905-s001.zip › foods-3289700-supplementary.pdf]

Table S1. Antimicrobial Properties of EOs derived from aromatic plants against the most important Gram-negative poultry meat isolates.

Family	Species	Plant part	EOs extraction method	Dose	Gram-negative bacterial strains	Results (MIC, MBC, IC_{50})	Mechanism of action	References
Lauraceae	*Cinnamomum cassia*	Bark	Hydro-distillation	1× MIC and 2× MIC	*Salmonella typhimurium, E. coli*	*S. typhimurium* (MIC 10 mg/mL, MBC 20mg/mL); *E. coli* (MIC 10 mg/mL, MBC 10 mg/mL)	disrupting the bacterial cell membrane	[108]
Lamiaceae	*Origanum vulgare*	Aerial parts	Hydro-distillation	-	*E. coli, Pseudomonas aeruginosa, Salmonella enterica*	MIC 0.16 %, MBC 0.16 %; MIC 0.63 %, MBC 1.25 %; MIC 0.08 %, MBC 0.08 %	-	[119]
-	Thymol (Roth), carvacrol, eugenol, guaiacol, and trans-anethol (Sigma-Aldrich)	-	-	The final amounts of tested compounds applied to the discs were 0.1, 0.4, 1, and 4 mg	*E. coli, Pseudomonas aeruginosa*	After 3 days of exposure, thymol reduced the amount of biofilm of *Pseudomonas aeruginosa* within the range of 70–77%. Carvacrol inhibited the formation of biofilms by up to 74–88% for *Pseudomonas aeruginosa*. Those phenols decreased the enzyme activity of the biofilm by up to 40–100%. After 10 days of exposure to thymol, biofilm formation was reduced by 80–100% for *Pseudomonas aeruginosa*. Carvacrol reduced the amount of biofilm by up to 91–100% for *Pseudomonas aeruginosa*	inhibition biofilm formation	
Lamiaceae	*Rosmarinus officinalis*	Leaves	Steam distillation	0.2%, 0.3%, 0.5% and 1.0%	*Salmonella Typhimurium, Listeria monocytogenes*	-	preventing growth of food-borne pathogens	[126]
Lamiaceae	*Rosmarinus officinalis*	Leaves and flowers	Hydro-distillation	5.0 mg/ml	*Salmonella enterica* subsp. enterica serovar Enteritidis	5.0 mg/mL	reduced bacterial population	[127]
Lamiaceae	*Ocimum basilicum*	Aerial parts	Hydro-distillation	2.5 mg/ml	*Salmonella enterica* subsp. enterica serovar Enteritidis	2.5 mg/mL	reduced bacterial population	[127]
Lamiaceae Lamiaceae Rutaceae	*Thymus vulgaris, Origanum vulgare, Citrus limonum*		Obtained in pure form	0.5 and 1%	*Salmonella typhimurium*	-	reduction of *S. typhimurium* counts with highest inhibition obtained using 1% lemon EO	[128]
Lamiaceae	*Thymus vulgaris and Origanum vulgare*	-	-	0.01 mL, 0.02 mL, 0.05 mL, 0.1 mL	*Salmonella* spp.	Thyme EO (with an MIC of 0.5%–1.0%) demonstrated significant antimicrobial activity against *Salmonella*. Oregano EO did not show inhibitory effects at any concentration tested	-	[129]
Verbenaceae Lauraceae Poaceae Lauraceae Lamiaceae Myrtaceae	*Aloysia triphylla, Cinnamomum zeylanicum, Cymbopogon citratus, Litsea cubeba, Mentha piperita, Syzygium aromaticum*	-	-	*A. triphylla* 171 μg, *C. zeylanicum* 202 μg, *C. citratus* 179 μg, *L. cubeba* 177 μg, *M. piperita* 182 μg, *S. aromaticum* 211 μg and *C. zeylanicum* 101 μg and *S. aromaticum* 105 μg for the mixture	*S. Enteritidis, S. Thyphimurium*	*Aloysia triphylla* (MIC 17.1 mg/mL) *Cinnamomum zeylanicum* (MIC 0.63-1.26 mg/mL) *Cymbopogon citratus* (MIC 17.9 mg/mL) *Litsea cubeba* (MIC 8.85-17.7 mg/mL) *Mentha piperita* (MIC 9.12-18.24 mg/mL) *Syzygium aromaticum* (MIC 0.16-2.63 mg/mL)	-	[130]
-	Carvacrol	-	-	1, 5, 10, 15, 20, 30, 40 or 50 μl	*Salmonella enterica* serotype Enteritidis	Minimum conc. of 20% carvacrol v/v in ethanol was required to achieve a significant reduction and from 40% v/v no viable cells were recovered	inhibition of growth of *S. enterica* serotype Enteritidis	[131]
Myrtaceae Lamiaceae	*Melaleuca alternifolia Thymus vulgaris*	-	-	15 mg	*E. coli, Salmonella gallinarum*	MIC of Tea tree oil: 0.15% (*E.coli*); 0.07% (*S. gallinarum)* MIC of Thyme oil: 0.03% (*E.coli* and *S. gallinarum)*	-	[132]
Lamiaceae	*Satureja hortensis*	-	Clevenger apparatus	10 μl	*Escherichia coli O78:K80* and *Salmonella Enteritidis*	MIC: 0.07 to 0.15 μl/ml (*E. coli)*; 0.31 to 0.62 μl/ml (*Salmonella)* MBC: 0.15 (*E. coli)*; 0.625 μl/ml (*Salmonella*)	-	[133]
Apiaceae Ranunculaceae Myrtaceae Lauraceae	*Cuminum cyminum Nigella sativa Syzygium aromaticum Cinnamomum cassia*	-	-	50 mg/mL and 100 mg/mL (in dimethyl sulfoxide (DMSO) solvent).	*Escherichia coli O157: H7, Klebsiella pneumonia*	Inhibition zone ranged from 14mm to 45mm	-	[134]

Lamiaceae	*Origanum majorana*							
Verbenaceae<br>Burseraceae<br>Lauraceae<br>Rutaceae<br>Rutaceae<br>Rutaceae<br>Rutaceae<br>Poaceae<br>Myrtaceae<br>Lamiaceae<br>Lauraceae<br>Lamiaceae<br>Myrtaceae<br>Lamiaceae<br>Geraniaceae<br>Myrtaceae	*Aloysia tryphilla, Boswellia sacra, Cinnamomum zeylanicum, Citrus aurantium, Citrus bergamia, Citrus limon, Citrus reticulata, Cymbopogon citratus, Eucalyptus globulus, Lavandula hybrida, Litsea cubeba, Ocimum basilicum, Melaleuca alternifolia, Mentha piperita, Pelargonium graveolens, and Syzygium aromaticum*	-	-	EOs were generally diluted with dimethyl sulfoxide (DMSO) for the antibacterial testing (a 1:10 dilution), and the MIC tests were carried out using the broth microdilution method, starting at a dilution of 10% (v/v) for antibacterial tests	*E. coli, Aspergillus fumigatus*	*E. coli* MIC 1.14-17.8 mg/ml;<br>*Aspergillus fumigatus* MIC 0.855- >26.35 mg/mL	-	[135]
Lamiaceae<br>Lamiaceae<br>Myrtaceae	*Thymus vulgaris*<br>*Rosmarinus officinalis*<br>*Syzygium aromaticum*	*The aerial parts*	Hydro-distillation method with Clevenger apparatus	1.5%	*E. coli*	Inhibition zone for<br>*Thymus vulgaris* 12.50mm<br>*Rosmarinus officinalis* 9.50mm<br>*Syzygium aromaticum* 14.50mm	-	[136]
Lamiaceae<br>Lamiaceae	*Rosmarinus officinalis*<br>*Mentha piperita*			0.5%	*E. coli*	Inhibition zone ranged from 9.00-15.43mm	-	[137]
Lamiaceae	*Thymus vulgaris*	-	-	0.4 mg/mL	*Campylobacter jejuni*	Free EO reduced the *C. jejuni* population by 2.16 log CFU/mL within 24 hours, but this effect was short-lived due to essential oil's instability and volatilization	disruption of the bacterial cell membrane	[138]
Lamiaceae	*Thymus vulgaris*<br>*Coriandrum sativum*	-	-	1% and 2%	*Campylobacter jejuni*	Thyme oil (1 and 2%) decreased count of *C. jejuni* (cfu/g) from 3.8x107 (initial load) to 7.3x105, and 1.2 x103 with reduction percentages 97.27% and 99.99% on 6th day of storage, respectively. Coriander oil (1 and 2%) decreased count of *C. jejuni* (cfu/g) to 3.8x106 and 9.5 x105 with reduction percentages 85.00% and 96.27% on 6th day of storage, respectively	-	[143]
Poaceae<br>Lauraceae<br>Geraniaceae<br>Myrtaceae<br>Lamiaceae<br>Myrtaceae<br>Santalaceae<br>Poaceae<br>Fabaceae<br>Alliaceae<br>Lamiaceae	*Cymbopogon Citratus, Cinnamomum zeylanicum, Pelargonium graveolens, Syzygium aromaticum, Origanum vulgare, Melaleuca alternifolia, Santalum austocaledonicum, Cymbopogon nardus, Thymus vulgaris, Copaifera langsdorffii, Allium sativum, and Lavandula angustifolia*	-	-	35 µL	*Campylobacter jejuni, Campylobacter coli*	Formulations with essential oils show MIC values ranging from 0.19 to 0.78 mg/mL for most *Campylobacter* strains, except for certain *C. jejuni* strains where MIC values increase, reaching up to 39.47 mg/mL	-	[144]
